# Use of biotics in animals: impact on nutrition, health, and food production

**DOI:** 10.1093/jas/skaf061

**Published:** 2025-02-27

**Authors:** Kelly S Swanson, Karin Allenspach, Gregory Amos, Thomas A Auchtung, Shalome A Bassett, Charlotte R Bjørnvad, Nadia Everaert, Susana M Martín-Orúe, Steven C Ricke, Elizabeth P Ryan, George C Fahey

**Affiliations:** Department of Animal Sciences, University of Illinois Urbana-Champaign, Urbana, IL 61801, USA; Department of Pathology, College of Veterinary Medicine, University of Georgia, Athens, GA 30602, USA; Waltham Petcare Science Institute, Melton Mowbray LE13, UK; Synbiotic Health, Lincoln, NE 68508, USA; Fonterra Limited, Fonterra Research & Development Centre, Palmerston North 4442, New Zealand; Riddet Institute, Massey University, Palmerston North 4442, New Zealand; Department of Veterinary Clinical Sciences, University of Copenhagen, Copenhagen, Denmark; Department of Biosystems, KU Leuven, Leuven, Belgium; Department of Animal and Food Science, Animal Nutrition and Welfare Service, Universitat Autònoma de Barcelona, Bellaterra, Spain; Department of Animal and Dairy Sciences, Meat Science and Animal Biologics Discovery Program, University of Wisconsin-Madison, Madison, WI 53706, USA; Department of Environmental and Radiological Health Sciences, Colorado State University, Fort Collins, CO 80524, USA; Department of Animal Sciences, University of Illinois Urbana-Champaign, Urbana, IL 61801, USA

**Keywords:** animal health, antimicrobial resistance, food safety, gastrointestinal health, immune modulation

## Abstract

Probiotics, prebiotics, and other biotic substances are not only effective ways to promote a healthy gastrointestinal tract, an effective immune system, and the overall health of humans, but also in agricultural and companion animals. Because key differences exist in regard to gastrointestinal tract anatomy and physiology, dietary management and feeding strategy, and disease susceptibility, however, biotic types and amounts often differ according to host species and life stage. Despite these differences, the literature demonstrates the value of biotics in agricultural and companion animal species. While high variability in responsiveness and efficacy has been reported, biotic substances may be effectively used to improve digestion, reduce morbidity, increase growth rate and/or efficiency in agricultural animals and promote gastrointestinal health and immune response in companion animals. As the oversight of antibiotic use intensifies, the population density of animals and humans increases, and production strategies of agricultural animals are more heavily scrutinized, the importance of biotics and other health promotors will continue to increase in the future. To date, the effects of animal biotic use have focused primarily on the farm, home, or veterinary clinic. In the future, their impact must be viewed on a larger scale. As global “One Health” approaches seek to reduce antimicrobial use and resistance and there are increasing demands for sustainable and safe food production, biotics will continue to be an important part of the solution. As knowledge of gastrointestinal microbiomes grows and the biotic field develops, more targeted and effective strategies for health promotion in these species are expected. At the 2023 International Scientific Association for Probiotics and Prebiotics meeting, experts were invited to participate in a discussion group focused on “The Use of Probiotics and Prebiotics in Agricultural and Companion Animals”. This review reports the outcomes of that discussion, including the documented use of probiotics, prebiotics, and other biotic substances to promote health or treat disease in agricultural and companion animals, provide implications of animal biotic use on human health, and provide perspective on how scientific advances may impact the development and improvement of biotics in the future.

## Teaser

Probiotics, prebiotics, and other biotic substances are effectively used to improve the health and well-being of agricultural animals and companion animals. At the 2023 International Scientific Association for Probiotics and Prebiotics meeting, experts were invited to participate in a discussion group focused on “The Use of Probiotics and Prebiotics in Agricultural and Companion Animals”. This review reports the outcomes of that discussion, including the successful use of animal biotics, implications of animal biotic use on human health, and perspective on how scientific advances may impact future biotic development and improvement.

## Introduction

Probiotics (Hill et al., 2014), prebiotics (Gibson et al., 2017), synbiotics (Swanson et al., 2020), and postbiotics (Salminen et al., 2021) are used to promote the health of agricultural and companion animals. In agricultural animals, biotic substances have been shown to increase nutrient digestion, reduce morbidity and mortality, reduce stress-related behaviors, and increase growth rate and/or efficiency. In companion animals, biotics have been shown to maintain or improve gastrointestinal tract (**GIT**) health outcomes, promote immune response, and improve metabolic health. Similar to humans, lactic acid bacteria (e.g., *Lactobacillus, Enterococcus, Pediococcus*), *Bifidobacterium*, and related microbial taxa have served as the most common probiotics in animal hosts. The most common prebiotics used in animals include fructans [e.g., inulin, oligofructose, short-chain fructooligosaccharides (**scFOS**)] and galactooligosaccharides. Other prebiotic candidates such as arabino-, mannan-, isomalto-, and xylooligosaccharides, beta-glucans, and unrefined prebiotic sources and yeast- and bacteria-based postbiotics are also commonly used.

Animal biotic characteristics and expectations of efficacy are similar to those of human products in many ways. However, the format in which they are provided (in food/feed), the conditions in which they are stored (hot temperatures; high humidity), and their interactions with other dietary ingredients when manufacturing complete and balanced diets may be quite different. Probiotics must not only be resistant to gastric acid, bile, enzymes, and other host secretions but must remain viable during food/feed storage and processing to be effective. Similarly, prebiotics and postbiotics must be stable during processing and food/feed storage so that they remain resistant to hydrolysis or absorption by the host to deliver bioactive substrates to the hindgut (Swanson et al., 2020).

When comparing animal host species, there are many host differences in GIT anatomy, physiology, microbiota populations, dietary requirements, and disease susceptibility that must be considered (Yeoman and White, 2014; Barko et al., 2018). The feeding strategies, housing conditions, hygiene levels, and environmental exposures also are quite different depending on the host species. Therefore, response to biotics is dependent on dietary nutrient composition, the resident microbiome, and other factors such as genetics, age, and living environment (Maldonado-Gomez et al., 2016; Whelan et al., 2024). The primary goals of raising agricultural animals and companion animals differ and must be considered. The goals of raising agricultural animals usually pertain to pathogen or disease resistance, rapid growth, and efficient and safe food production. In contrast, primary concerns for companion animals relate to health and longevity and may include supporting gastrointestinal health, maintaining health in geriatrics, preventing or managing obesity, improving performance during exercise and work, and alleviating stress. Despite the differences among host species, biotic administration early in life to prevent pathogen colonization is a commonality. Later in life, the animal’s physiological status, disease status, management strategy, and other factors dictate whether or not biotic administration is relevant and by what means.

The scientific literature demonstrates a long history of successful biotic use in agricultural (Table 1) and companion animal (Table 2) species, but the field is ever-changing. There is room for improvement and expansion, for instance, as high variability in responsiveness and efficacy have been reported. As knowledge of animal gastrointestinal microbiomes grows and the biotic field develops, more targeted and effective strategies for health promotion in these species are expected. Biotics also should be included in strategies to tackle challenges such as antimicrobial resistance (**AMR**) and safe food production. While the focus of animal biotic use often has been on the farm, in the home, or at the veterinary clinic on a micro level, assessing and/or predicting the impact of biotics on a larger level must be included in the future.

**Table 1.** Examples of efficacy using biotics in agricultural animals

Animal species	Biotic treatment	Primary beneficial outcomes	Reference
*Swine*			
Piglets (2-d old) exposed to *S. typhimurium*	Soy polysaccharides or FOS (7.5 g/L) in liquid formula; 14-d intervention	Maintained stool consistency; increased glutamine transport by 10% to 20%	Correa-Matos et al., 2003
Piglets (16-d old)	Chitooligosaccharides (100, 200, or 400 mg/kg of diet); 21-d intervention	Reduced *E. coli* shedding (3.38 vs. 3.87 log cfu/g of wet digesta); increased growth (315 g/d vs. 285 g/d) and efficiency (gain:feed of 0.69 vs. 0.66); reduced diarrhea incidence (5.7% vs. 13.8%) and severity (score of 8 vs. 19)	Liu et al., 2008
Piglets (3-wk old) infected with porcine reproductive and respiratory syndrome virus	Mannanoligosaccharides (0.2% of diet) supplementation; 4-wk intervention	Reduced fever; increased feed efficiency (gain:feed of 0.55 vs. 0.50) and immune function (white blood cell and lymphocyte counts)	Che et al., 2011
Piglets (24-d old)	*Lactobacillus reuteri* P7, *L. amylovorus* P8, and *L. johnsonii* P15 in feed (2 × 10^8^ CFU/g); 4-wk intervention	Increased growth (approximately 400 g/d vs. 275 g/d) and efficiency (feed:gain of 1.7 vs. 2.1); decreased diarrhea incidence (5% vs. 20%); increased antioxidant capacity	Wang et al., 2021
Piglets (2-d old)	*Saccharomyces cerevisiae var. boulardii* CNCM-1079 or *Enterococcus faecium lactiferm* WS200 (1 × 10^10^ CFU); single dose	Increased growth (251 g/d for *E. faecium*, 246 g/d for *S. cerevisiae*, and 212 g/d for control); reduced mortality (1.4% for *S. cerevisiae* vs. 3.9% for controls); modulated microbiota	Luise et al., 2021
Piglets (28-d old) challenged with enterotoxigenic *E. coli* F18	Multi-species probiotic (4 × 10^9^ CFU/pig); 5-wk intervention	Reduced enterotoxigenic *E. coli* shedding (13/20 piglets vs. 21/24 piglets); reduced heat-stable enterotoxin b in feces	Hansen et al., 2022
*Poultry*			
Broiler chicks (1-d old)	Inulin (5, 10, 15, and 20 g/kg of diet); 5-wk intervention	Increased Ca (up to 19% higher), Zn (up to 36% higher), and Cu (up to 467% higher) retention; increased bone density (5-6% greater)	Ortiz et al., 2009
Broiler chicks (1-d old) subjected to heat stress	Mannanoligosaccharides (0.5% of diet); 42-d intervention	Increased growth rate (1.9 vs. 1.6 kg over 42 d) and efficiency (feed:gain of 1.39 vs. 1.67)	Sohail et al., 2012
Broiler chick embryos (day 12 of incubation period)	Galactooligosaccharides (0.18, 0.88, 3.5, or 7.0 mg/embryo); single dose	Increased growth rate (855 g vs. 784 g over 3 wk)	Bednarczyk et al., 2016
Laying hens (40-wk old)	*Enterococcus faecium* DSM 7134 (0.005 or 0.010% of diet); 27-wk intervention	Increased egg production (97.8% vs. 96.6%); increased egg shell thickness (39.2 vs. 38.0 mm); increased dry matter (83.9% vs. 78.0%) and nitrogen (73.9% vs. 70.5%) digestibility; reduced excreta ammonia emissions (21.6 vs. 73.3 ppm)	Park et al., 2016
Broiler chicks (1-d old)	*Lactobacillus reuteri* and *Streptomyces coelicolor (S. coelicolor)* (50 or 100 mg/kg diet); 8-wk intervention	Increased growth rate and efficiency, with efficacy depending on week	Bhogoju et al., 2021
Laying hens (30-wk old)	*Clostridium butyricum* zlc-17, *C. butyricum* lwc-13, or *Brevibacillus* zlb-z1 (0.02% of diet); 84-d intervention	Increased protein and amino acid digestibilities and feed efficiency (feed:gain of 1.84 vs. 1.97); increased eggshell thickness (48.6 vs. 46.9 mm); increased immune function (greater white blood cell and lymphocyte counts)	Obianwuna et al., 2022
*Ruminants*			
Pre-ruminant calves (7-d old)	Galactosyl-lactose (1% of dietary dry matter); 26-d intervention	Increased growth rate (197 vs, 125 g/d); reduced number of d (16.9 vs. 19.4 d) and severity of scouring (fecal scores of 2.06 vs. 2.27)	Quigley et al., 1997
Pre-ruminant calves (40 kg BW)	Multistrain probiotic (7 bacterial and 2 yeast strains; 2 × 10^9^/d), *Saccharomyces cerevisiae* cell wall polysaccharide (4 g/d), or probiotic-cell wall polysaccharide combination; 60-d intervention	Increased growth rate (1.1, 1.2, and 1.3 vs. 0.97 kg/d during week 8); reduced fecal shedding of *E. coli* (7.0, 7.1, and 7.4 vs. 7.6 log cfu/g of wet digesta)	Roodposhti and Dabiri, 2012
Dairy cows (10 d prepartum)	Direct-fed microbial (2 *Enterococci faecium* strains and *Saccharomyces cerevisiae*, each at 5 × 10^9^ CFU, 2 g/cow per d); 33-d intervention	Increased milk fat percentage for first lactation cows (3.6% vs. 3.3%); increased milk protein percentage for second and greater lactation cows (3.13% vs. 3.10%)	Oetzel et al., 2007
Dairy cows (2 wk prepartum)	Weekly intravaginal infusion of *Lactobacillus sakei* and *Pediococcus acidilactici* strains (10^8^ to 10^9^ CFU/dose); 3-wk intervention	Increased feed efficiency and milk production; improved measures of reproduction	Deng et al., 2016
Beef steers (360 kg BW)	*Lactobacillus acidophilus* NP51 (10^9^ CFU/d);18 wk period	Reduced fecal shedding of *E. coli* O157:H7	Peterson et al., 2007
Lambs (90-d old)	Mixture of *Lactobacillus casei* HM-09 (1.5 × 10^9^ CFU/g) and of *Lactobacillus plantarum* HM-10 (1.5 × 10^9^ CFU/g) at 10 g/d; 90-d intervention	Increased muscle production and improved meat quality (lower pH: 5.41 vs. 5.77; lighter color: 33.69 vs. 35.20 L*; lower shear force: 71.8 vs. 79.3 Newtons)	Liu et al., 2022a
*Aquaculture*			
Caspian roach (*Rutilus rutilus caspicus*) fry (approximately 1.4 g BW)	Galactooligosaccharides (1 or 2% of diet); 7-wk intervention	Increased survival rate (93.5% vs. 74.8%); increased growth rate (237% vs. 192% body weight over 7 wk); increased resistance to salinity stress challenge (72% vs. 55% survival)	Hoseinifar et al., 2013
Common carp (*Cyprinus carpio*) fry (approximately 3.2 g BW)	Fructooligosaccharides (1, 2, or 3% of diet); 7-wk intervention	Increased immune function and survival rate (up to 98.3% vs. 61.6% in controls); increased stress resistance to salinity stress challenge (up to approximately 60% vs. approximately 35% in controls)	Hoseinifar et al., 2014
Fringed-lipped peninsula carp (*Labeo fimbriatus*) fry (approximately 3.9 g BW)	Fructooligosacharides (0.5% of diet), *Bacillus subtilis* (10^4^ or 10^6^ CFU/g diet), or combinations; 60-d intervention	*Bacillus subtilis* increased growth rate (up to approximately 0.85 specific growth rate vs. approximately 0.35 in controls) and efficiency (down to approximately 1.8 feed:gain vs. approximately 2.6 in controls); both interventions increased immune function and pathogen (*Aeromonas hydrophila* O:18) challenge survival (up to approximately 90% at 7 d post-challenge vs. approximately 45% in controls)	Pawar et al., 2023

**Table 2.** Examples of efficacy using biotics in companion animals

Animal species	Biotic treatment	Primary beneficial outcomes	Reference
*Dogs*			
Healthy adult dogs (beagles)	Cellulose (6% of diet), fructooligosaccharides (1.5% of diet), beet pulp (6% of diet), or fiber blend (6% beet pulp, 2% gum arabic, and 1.5% fructooligosaccharides); 35-d intervention	Fiber blend increased fecal nitrogen (2.1 vs. 1.6 g/d) and microbial nitrogen excretion (0.55 vs. 0.37 g/d); reduced urinary nitrogen excretion [4.8 (fructooligosaccharides), 6.0 (beet pulp) and 6.7 (fiber blend) g/d vs. 7.7 g/d in controls]	Howard et al., 2000
Hound-cross puppies (12 wk old) infected with *Salmonella typhimurium* DT104	Short-chain fructooligosaccharides (1% of diet); 14-d intervention	Reduced enterocyte sloughing (pathology score of 0.4 vs. 1.2 in controls); reduced fever; maintained intestinal glucose transport (1.7 Isc/cm^2^ vs. 0.6 Isc/cm^2^ in controls)	Apanavicius et al., 2007
Obese adult dogs (beagles)	Short-chain fructooligosaccharides (1% of diet); 6-wk intervention	Increased insulin response (7.7 mg/kg/min glucose infusion rate vs. 4.7 mg/kg/min in controls); modified adipose tissue mRNA expression of genes associated with fatty acid and glucose metabolism	Respondek et al., 2008b
Young adult dogs diagnosed with acute idiopathic diarrhea	*Bifidobacterium animalis* AHC7 (2 × 10^10^ CFU/d); 14-d intervention	Reduced number of days to resolution of diarrhea (3.9 vs. 6.6 d)	Kelley et al., 2009
Hospitalized adult dogs diagnosed with acute gastroenteritis	Probiotic paste (2.85 billion live strains of *Lactobacillus farciminis*, *Pediococcus acidilactici*, *Bacillus subtilis*, and *Bacillus licheniformis*, and 1.35 billion thermo-stabilized *Lactobacillus acidophilus* per mL), with dose based on BW (1-10 kg: 1 mL; 10-25 kg: 2 mL; 25 to 50 kg: 3 mL); given 3 times daily, starting with a double dose, until normalization of stools	Reduced number of days to normalization of stool (1.3 vs. 2.2 d)	Herstad et al., 2010
Boxer puppies (60 d old) infected with enteropathogenic *E. coli*	Mannanoligosaccharides (2 g/kg BW once daily); 20 d	Faster recovery (normalized feces)	Gouveia et al., 2013
Dogs entering an animal shelter	Combination of probiotic (*Enterococcus faecium* NCIMB 10415 4b1707, 2 × 10^9^ CFU/capsule) and prebiotic (fructooligosaccharides and gum arabic, 46.4 mg/capsule); 1 capsule/d	Reduced incidence of diarrhea during stay (2.0% vs. 3.2%)	Rose et al., 2017
Adult dogs (beagles) fed high-fat diet	*Enterococcus faecium* IDCC 2102 (1 × 10^10^ CFU/d) and *Bifidobacterium lactis* IDCC 4301 (1 × 10^10^ CFU/d); 9 wk intervention	Limited weight gain (15.4% or 17.3% lower final BW); attenuated increases in blood insulin, leptin, and inflammatory cytokines	Kang et al., 2024
*Cats*			
Cats in an animal shelter	*Enterococcus faecium* SF68 (2.1 × 10^9^ CFU/d); 4 wk intervention	Reduced incidence of diarrhea > 2 d (7.4% vs. 20.7%)	Bybee et al., 2011
Fostered shelter kittens (< 12 wks old)	*Enterococcus hirae* strain 1002-2 (1 × 10^8^ CFU/d)	Reduced incidence of diarrhea (3.4 times less likely)	Gookin et al., 2022
*Horses*			
Horses with acute enterocolitis	*Saccharomyces boulardii* (10 × 10^9^ or 20 × 10^9^ CFU every 12 h based on BW); 14-d intervention	Reduced severity and duration of illness (5 vs. 7 d of diarrhea)	Desrochers et al., 2005
Obese adult horses	Short-chain fructooligosaccharides (45 grams/d); 6-wk intervention	Increased insulin sensitivity [fasting insulin: 14.4 vs. 24.1 mU/L; acute insulin response to glucose: 577 vs. 863 (mU·min)/L]	Respondek et al., 2011
Adult horses undergoing exercise challenge	Probiotic mixture (*Lactobacillus acidophilus* DSM 32241, *Lactobacillus plantarum* DSM 32244, *Lactobacillus casei* DSM 32243, *Lactobacillus helveticus* DSM 32242, *Lactobacillus brevis* DSM 27961, *Bifidobacterium lactis* DSM 32246, *Bifidobacterium lactis* DSM 32247, and *Streptococcus thermophilus* DSM 32245), 35 g/d; 21-d intervention	Modified blood and urinary metabolite profiles; reduced post-exercise blood lactate concentrations (approximately 10 mmol/L vs. approximately 14 mmol/L)	Laghi et al., 2018
Healthy adult horses subjected to transport and exercise stress	*Aspergillus oryzae* fermentation product (0.02 g/kg BW); 28 d prior to transport/exercise stress	Prevented gastrointestinal hyperpermeability (leaky gut)	McGilloway et al., 2023b

At the 2023 International Scientific Association for Probiotics and Prebiotics (**ISAPP**) meeting, experts were invited to participate in a discussion group focused on “The Use of Probiotics and Prebiotics in Agricultural and Companion Animals”. This review reports the outcomes of that discussion, including (1) the documented use of probiotics, prebiotics, and other biotic substances to promote health or treat disease in agricultural and companion animals, (2) a description of how animal biotic use may extend to and impact human health, and (3) propose how technological advances may shape the future of the field.

## Importance of early life microbial colonization

Early life GIT maturation comprises structural and functional changes that progress with age, affecting digestive and absorptive capacities. Microbial colonization is an important contributor to GIT maturation, immune system development, pathogen resistance, and overall host health. Once mammalian species are born and birds hatch, the GIT undergoes a successive colonization process, initially by facultative aerobes and later by obligate anaerobes. A similar process occurs when fish species hatch or are born. Colonization is influenced by exposure to microorganisms from the mother’s vagina during parturition, the rearing environment, and feed and water (Sekelja et al., 2012). The microbial colonization process has been described for chicks (Ballou et al., 2016; Jurburg et al., 2019), piglets (Liu et al., 2019), calves (Du et al., 2023), horses (De La Torre et al., 2019), dogs (Del Carro et al., 2022), cats (Hooda et al., 2013; Deusch et al., 2015), and several fish species (Stephens et al., 2016; Deng et al., 2021; Lokesh et al., 2024). It is well known that the GIT microbiota of young growing animals have a lower stability and diversity than adults, making them more susceptible to stress or challenge.

Active substances in colostrum and milk, such as antibodies and milk oligosaccharides, affect the intestinal microbiota of neonates, favoring the development of beneficial bacteria (e.g., *Lactobacillus*) and preventing pathogen growth (Tian et al., 2019). Mammals are born hypogammaglobulinemic and must acquire passive immunity through antibodies such as immunoglobulin (Ig) G via colostrum and/or milk once born and nursing. The transfer of maternal IgY from the yolk to the chick starts at the end of the first week of embryonic development (Härtle et al., 2022). The host innate immune system (e.g., beta-defensins, small antimicrobial peptides present on intestinal epithelial surfaces) interacts with the GIT microbial community, leading to subsequent adaptive immune responses. The presence of microbiota also affects goblet cell number and density, mucin expression, and other physiological responses (Cheled-Shoval et al., 2014).

Weaning stress, due to a plethora of social, dietary, and environmental changes, can predispose young animals to dysbiosis, characterized by lower microbial diversity and increased abundance of facultative anaerobes, including bacteria belonging to the *Enterobacteriaceae*, *Proteobacteriaceae*, *Clostridiaceae,* and *Prevotellaceae* families (Gresse et al., 2017). As animals mature, GIT microbial species richness and microbiota population complexity increase, eventually reaching a state of maturity and stability. Environmentally enriched housing or inoculation with commensal microbes may drive maturation of the GIT microbiota, reduce pathogen load, and improve immune competence (Redweik et al., 2020; Wen et al., 2021). Understanding the intricate relationships among morphological, functional, and immunological development, which is shaped by both initial microbial colonization and the ingestion of dietary elements, is crucial for pinpointing effective approaches to enhance intestinal health through management and feeding strategies (Everaert et al., 2017).

## Use of biotics in agricultural animals

### Swine

Biotic strategies have been used in swine production for decades (Barba-Vidal et al., 2019), but the biotic concept has evolved substantially over time. Because environmental stressors and pathogen loads are high in young piglets, the use of biotics was initially viewed as an alternative to antimicrobials, providing antimicrobial peptides (Sanca et al., 2023) or enzymes (Shen et al., 2022) to improve digestion. Recent knowledge of the GIT microbiota and its pivotal role in animal health has led to a paradigm shift whereby biotic strategies are used to improve animal health and performance.

Probiotics are a powerful tool to reduce the need of using antimicrobials (Cardelle-Cobas et al., 2022), inhibiting pathogens such as enterotoxigenic *Escherichia coli* K88 (Wang et al., 2021), *Brachyspira* (Bernardeau et al., 2009), and *Lawsonia intracellularis* (Muwonge et al., 2021). Probiotic anti-pathogen activity has been associated with the down-regulation of virulent genes and disruption of quorum-sensing (Sudan et al., 2021) and the production of antimicrobial peptides or structural components such as lipoteichoic acids, teichoic acids, peptidoglycans, or surface-layer proteins that also apply to postbiotic strategies (Ali et al., 2023). Improved growth rate and efficiency, greater ingredient digestion and nutrient absorption, reduction in incidence of diarrhea and fecal *E. coli* shedding, greater immune response, and reduced infection-associated responses to *Salmonella typhimurium* or porcine reproductive and respiratory syndrome virus have been reported with prebiotic [fructooligosaccharides, mannanoligosaccharides (MOS), chitooligosaccharides, or soy polysaccharides] supplementation in young pigs (Correa-Matos et al., 2003; Liu et al., 2008; Che et al., 2011; Halas and Nochta, 2012).

Many of the improvements reported in digestive function and performance may be attributed to the positive impact of probiotics on the GIT microbiota. In this regard, early age represents a valuable window of opportunity for intervention. Early probiotic inoculation may prevent dysbiosis and post-weaning diarrhea in piglets (Luise et al., 2021; Hansen et al., 2022). Inoculating the sow (Saladrigas-García et al., 2022) or adding biotics to creep feeds may also be used to accelerate GIT microbiota maturation and reduce post-weaning diarrhea (Choudhury et al., 2021). The use of probiotics and/or prebiotics also has been proposed as a way to restore the microbiota after being disturbed by pathogens and/or the use of antimicrobials (Jo et al., 2021). Because detrimental behaviors such as tail biting are related to dysbiosis (Kobek-Kjeldager et al., 2022), biotic strategies also may reduce stress, improve behavior, and improve welfare of pigs (Choudhury et al., 2022). Finally, biotics may be used to improve pork meat quality, modulate adipogenesis and myogenesis (Kim et al., 2022), increase inosine concentration (Alba et al., 2023), and improve longissimus muscle area, loin weight, and marbling or juiciness scores (Lee et al., 2021).

### Poultry

The primary objectives for employing biotics in poultry production are performance enhancement and ensuring food safety (Clavijo and Flórez, 2018). Over the past few decades, substantial research efforts have been dedicated to screening various biotic substances to assess their economic benefits in poultry production (Patterson and Burkholder, 2003). For broilers, improved performance based on increased body weight gains and improved feed conversion has been reported in some studies (Bhogoju et al., 2021; Li et al., 2021). The desired outcomes for egg-laying hens involve enhanced egg quality, increased egg production, and decreased excreta ammonia emissions (Park et al., 2016; Peralta-Sánchez et al., 2019; Obianwuna et al., 2022). Notably, both prebiotics and probiotics have been widely utilized to reduce the colonization of foodborne pathogens, particularly *Salmonella* spp. and C*ampylobacter jejuni*, in the GIT of poultry (Clavijo and Flórez, 2018).

Prebiotics (inulin, MOS, lactulose) and postbiotics (yeast cell product) may improve growth performance, gastrointestinal morphology, immune responsiveness to experimental coccidial infection, response to heat stress, and mineral retention and bone mineralization that have beneficial effects on bone and egg quality (Ortiz et al., 2009; Sohail et al., 2012; Shanmugasundaram et al., 2013; Cho and Kim, 2014). Unique to birds, prebiotics may not only be delivered in the drinking water or diet but also in ovo prior to hatching (Roto et al., 2016). In ovo injections of prebiotics and synbiotics maintain hatchability success and increase pancreatic enzyme activity and weight gain (Pruszynska-Oszmalek et al., 2015; Bednarczyk et al., 2016).

Several mechanisms underlie the effectiveness of prebiotics and probiotics in poultry, including the production of antagonistic short-chain fatty acids (**SCFA**), competitive exclusion, modulation of the GIT microbiota, and immunomodulation, among others (Adhikari and Kim, 2017; Teng and Kim, 2018). Traditionally, early administration of probiotics to prevent the establishment of *Salmonella* in young chicks has been considered vital to successful rearing. More recent evidence suggests that even earlier in ovo application prior to hatching may be effective (Roto et al., 2016; Siwek et al., 2018).

Recent microbiome analyses of the poultry GIT have revealed a more complex response to prebiotics than originally thought (Ricke, 2018). Prebiotics and probiotics in poultry have traditionally focused on cecal activities, as this is the primary site of microbial carbohydrate fermentation leading to the production of SCFA that can inhibit pathogens (Józefiak et al., 2004). It is becoming evident, however, that the upper part of the avian GIT, including the crop and the small intestine, also may play a significant role in biotic efficacy. The crop serves as a primary site for lactobacilli, and studies on the mucosal lining of the small intestine have revealed that different *Lactobacillus* species populate different sections of the small intestine (Classen et al., 2016; Adhikari and Kwon, 2017). The presence of these distinct populations in various compartments of the poultry GIT may influence the overall GIT response to different prebiotics and the populations they enrich. In future poultry studies involving the commercial application of prebiotics and probiotics, standardizing microbiome analyses will be essential to allow for more accurate comparisons across studies and to establish common factors (Weinroth et al., 2022).

### Ruminants

As with other agricultural animals, the use of biotics in ruminants has been viewed as a potential solution to reduce antimicrobial use, primarily because of their immune health benefits, protection against gut pathogens, and GIT microbiome modulation. Ruminants are born in a pre-ruminant state and function as non-ruminants until the rumen and other compartments of the stomach (e.g., reticulum, omasum, abomasum) fully develop. Probiotic supplementation in pre-ruminants may increase average daily gain and feed efficiency (Saha et al., 2018; Hamdon et al., 2022; Wang et al., 2023). Probiotics may also improve immunity in calves, reducing the incidence of scours and maintaining growth rates during diarrheic episodes (Villot et al., 2019). Cellooligosaccharides, galactosyl-lactose, yeast cell wall extracts, and MOS improved weight gain and feed efficiency, reduced the severity and number of days scouring, and reduced *E. coli* shedding in calves (Quigley et al., 1997; Hasunuma et al., 2011; Ghosh and Mehla, 2012; Roodposhti and Dabiri, 2012; Baines and Erb, 2013).

The rumen is the major digestive organ of the ruminant. Consequently, any perturbation to the rumen can have significant impacts on the health, growth, and productivity of the animal. Increased nutrient digestibility improves growth and productivity measures in all animals, but is particularly important in ruminants that are fed roughages that are rich in dietary fiber. In adult ruminants, probiotic-mediated changes in the rumen may improve feed conversion efficiency, SCFA production, and nitrogen utilization, resulting in improved milk composition (e.g., fat and protein percentages) and milk or meat production (Nocek et al., 2003; Oetzel et al., 2007; Nalla et al., 2022). Dairy cows are a high-priority target because they often are fed high-grain diets that provide higher energy content, yet can lead to ruminal acidosis or trigger inflammatory-based diseases such as laminitis or mastitis. Probiotics that produce or utilize lactate are thought to help prevent sub-acute rumen acidosis by diminishing lactate concentrations and increasing rumen pH (Fenta et al., 2023).

Interestingly, oral administration of probiotics may not always be necessary. In one study, intravaginal infusions of a probiotic around parturition lowered uterine infection occurrence and improved local and systemic immune responses and the overall health status of periparturient dairy cows (Deng et al., 2015a). In other studies, intravaginal probiotic infusion resulted in fewer antimicrobial treatments (Oetzel et al., 2007), altered milk composition (e.g., protein, lactose), increased feed efficiency and milk production (Deng et al., 2016), and affected measures of reproduction (Deng et al., 2015b).

Probiotics also may improve meat quality and safety. In a beef steer study, probiotic supplementation reduced *E. coli* O157:H7 shedding (Peterson et al., 2007). Probiotic-supplemented lambs had improved meat tenderness (lightness and shear force), improved muscle fiber characteristics, altered volatile flavor compounds, and improved antioxidant capacity (Liu et al., 2022a). In a similar study, sheep fed a probiotic had altered blood lipids, expression of lipid metabolism-related genes, tail fat metabolites, and volatile flavor compounds (Liu et al., 2022b). Unlike some animal feeds, probiotic supplementation in ruminants has not been reported to cause residue issues in either meat or milk products or contribute to off-flavors.

### Aquaculture

Anatomy varies among carnivorous (e.g., turbot), omnivorous (e.g., catfish), and herbivorous (e.g., sturgeon) species, but all fish have a relatively simplistic and short GIT. Despite their simple GIT and short transit time, biotics have received much attention in recent years as regards their effects on the nutrition and health of select fish (e.g., tilapia, trout, salmon, catfish, red drum) and shellfish (e.g., shrimp, scallops, oysters) due to their potential influence on immunity and disease resistance (Gatlin and Yamamoto, 2021). As in other host species, most of the positive biotic effects in aquatic species are thought to be mediated by the host GIT where autochthonous bacteria provide a defensive barrier, protecting against bacterial, viral, and protozoal pathogens, and through the production of antimicrobial substances that minimize pathogen colonization (Gatlin and Peredo, 2012).

Many fish are raised in sub-optimal environments that have a substantial pathogen load. Administration of probiotics to the water has been shown to improve water quality (e.g., reduction in nitrogen and phosphorus concentrations) (Wang et al., 2005), inhibit the growth of pathogenic microorganisms, and stimulate host immune response. Probiotic administration also may provide digestive enzymes or growth-promoting factors that increase GIT absorptive area, improve nutrient and energy digestibility, and/or increase weight gain and feed efficiency.

Prebiotic (scFOS, galactooligosaccharides) supplementation may improve survival rate, growth rate, feed conversion ratio, immune responsiveness, and resistance to salinity stress or pathogen challenge (Li et al., 2007; Hoseinifar et al., 2013, 2014). MOS, which bind pathogenic microbes and eliminate them from the host, are also candidate prebiotics used in aquatic species (Gatlin and Peredo, 2012). Synbiotics also may have potential, but ideal prebiotic–probiotic combinations have yet to be identified. The application of dead cells, freeze-dried cells, or cell-free extracts or spores (known today as postbiotics) also have shown positive effects on nutrition and health outcomes in aquatic species (Merrifield et al., 2010; Sudhakaran et al., 2022), but more research is needed to determine their efficacy in aquatic species.

## Use of biotics in companion animals

### Dogs and cats

Prebiotics have been an integral part of pet food formulations for decades. In pets, prebiotics modulate fecal quality by modulating osmoregulation and reabsorption of water and electrolytes and reduce fecal odor through modulation of fermentation product formation and limiting bacterial putrefactive metabolism (Perini et al., 2023). Furthermore, prebiotics improve gastrointestinal conditions through complex modulation of the gut microbiota, SCFA production, and reduced colonic pH that ultimately improves intestinal barrier and immune function (Bosch et al., 2009; Pinna et al., 2018; Perini et al., 2023). In weanling puppies subjected to a *Salmonella typhimurium* challenge, inulin and scFOS supplementation maintained food intake and reduced the severity of enterocyte sloughing (Apanavicius et al., 2007). Similarly, a more rapid recovery from enteropathogenic *E. coli* infection was reported in dogs supplemented with phosphorylated MOS (Gouveia et al., 2013). Of relevance to pets with liver and renal disease, prebiotics such as FOS, inulin and a fiber blend (beet pulp, gum Arabic, and FOS) decrease circulating ammonia and urea by increasing fecal nitrogen excretion (Howard et al., 2000; Bosch et al., 2009; Pinna et al., 2018).

Another important area for use of prebiotics in pet food is weight management, improvement of glycemic control, and modulation of cholesterol metabolism. Prebiotic inclusion decreases the energy density of the diet, has been shown to slow glucose absorption, modulate sodium-glucose transporter 1 activity, and improve insulin response (Respondek et al., 2008b). A combination of sugar beet pulp and inulin tended to decrease voluntary food intake in dogs compared with a diet with low-fermentation fiber, possibly improving satiety by activating the ileal brake and decreasing gastric emptying through fermentation products (Bosch et al., 2009). There is variation in the individual tolerance and response to prebiotics among companion animals, with larger dogs generally having a lower tolerance. Additionally, response to prebiotic inclusion is dependent upon several factors (e.g., dietary nutrient composition, resident microbiome, individual factors such as genetics, age, and living environment). While prebiotics are a more likely strategy for controlling weight and metabolism, a probiotic treatment was recently reported to limit weight gain and improve metabolism in dogs fed a high-fat diet (Kang et al., 2024).

A recent systematic review (12 studies) assessed the evidence of probiotic efficacy for acute diarrhea cases in dogs (Jensen and Bjørnvad, 2019). While the authors concluded that there was limited evidence available, a few studies reported reduced duration of diarrhea with probiotic supplementation (Kelley et al., 2009; Herstad et al., 2010; Rose et al., 2017). Since then, 5 more canine randomized control trials assessing the clinical effects of probiotics have been published, including 3 comparing a probiotic against a placebo for acute diarrhea (Ziese et al., 2018; Nixon et al., 2019; Molina et al., 2023), one comparing a probiotic against metronidazole (Shmalberg et al., 2019), and one comparing a probiotic against fecal microbial transplantation (**FMT**) (Jugan et al., 2023). In 3 of those studies, no clinical effect could be discerned between the probiotic and placebo or FMT. In the 2 other publications, a mild effect of the probiotic was demonstrated by shortening the duration of acute diarrhea (Nixon et al., 2019; Molina et al., 2023). Further, in the recent systematic review and meta-analysis for the European network for Optimization of antimicrobial therapy guidelines, it was concluded that nutraceutical products (i.e., probiotics) did not show a clinically significant effect in shortening the duration of acute diarrhea (Scahill et al., 2024). Variance among studies may be due to differences in the probiotic administered (e.g., dosage; strains), the health status, age, and basal microbiome of the animals tested, and/or the cause of diarrhea.

The systematic review published by Jensen and Bjørnvad (2019) reported on 5 studies that tested probiotic efficacy in dogs with chronic diarrhea. The published evidence did not support the use of probiotic supplementation as standard treatment. Since then, 2 more randomized control trials testing probiotics in canine chronic diarrhea cases have been reported (White et al., 2017; Sahoo et al., 2022), with neither showing clinical improvement. Few studies have tested probiotic efficacy in cats with GI disease. In 2 studies, prophylactic probiotic supplementation has been shown to decrease the incidence of diarrhea in shelter cats (Bybee et al., 2011) and kittens (Gookin et al., 2022). In 2 other studies, no differences in clinical signs were observed in cats with antimicrobial-associated diarrhea (Torres-Henderson et al., 2017; Whittemore et al., 2019). Although many dog and cat studies have not had positive results, it is important to remember that currently, most probiotics do not originate from the host species they are treating. In the future, host-adapted probiotic strains that are active longer and have more ecologically relevant effects may have better outcomes.

### Equine

Horses are large non-ruminant herbivores that rely heavily on microbial fermentation for energy, with over half of their energy coming from microbial fermentation in their enlarged cecum and colon. In recent years, modulation of metabolic functions of the GIT microbiota by diet, probiotics, and prebiotics has received attention for clinically relevant applications. Yeast cell fermentation products and scFOS may increase nutrient digestibility of low-quality forage or reduce gastrointestinal pH and SCFA fluctuations in horses (Morgan et al., 2007; Respondek et al., 2008a). There is reported variability in response, however, that could be attributed to the strain, dose, and duration of administration, dietary composition, and host age and other physiological features. Dietary scFOS also has been demonstrated to improve insulin sensitivity in obese horses (Respondek et al., 2011). The magnitude of improvement is often associated with the degree of insulin resistance recorded at baseline and extent of body weight loss during the study, sometimes independent of scFOS supplementation (McGowan et al., 2013).

Probiotic strains and doses used in clinical trials to evaluate the effect of probiotics on gastrointestinal disease in horses have focused on reducing *Salmonella*-induced diarrhea, colic, and body weight, as reviewed by Schoster et al. (2014). Another common target of probiotic use has been young growing foals. The GIT microbiota population of foals has a lower diversity and stability than that of adult horses (De La Torre et al., 2019), making foals more susceptible to pathogen-induced microbiota alterations, diarrhea, dehydration, and intestinal inflammation (Schoster et al., 2017)

Probiotic use in foals has had both helpful and harmful outcomes. Positive results were obtained with a probiotic containing 5 *Lactobacillus* strains, which were shown to increase body weight and reduce diarrhea incidence in 3 to 4 wk old foals (Yuyama et al., 2004). Similarly, a probiotic composed of 4 *Lactobacillus* strains and 1 *Bifidobacterium* strain was reported to reduce the incidence and duration of diarrhea in foals during their first 5 mo of life (Tanabe et al., 2014). Administration of a different probiotic was associated with anorexia, development of diarrhea, and greater need for veterinary examination and treatment (Weese and Rousseau, 2005). Based on the evidence, caution should be used when considering probiotic use in foals.

In adult horses, GIT physiological and microbiota disruptions can occur with rapid changes in diet (Venable et al., 2017), transportation and/or exercise stress (Perry et al., 2018; McGilloway et al., 2023a), or the onset of GIT disease, laminitis, or grass sickness (Garrett et al., 2002; Costa et al., 2012; Moreau et al., 2014). Horses are susceptible to GIT disorders such as enterocolitis that may be due to antimicrobial use, stressful conditions, or pathogen infection (e.g., *Clostridioides difficile*; *Salmonella*). In one study, a *Saccharomyces boulardii* reduced the severity and duration of illness in horses with acute enterocolitis (Desrochers et al., 2005). In another study, a probiotic mixture of 3 *Lactobacillus* strains and 1 *Enterococcus* strain reduced the incidence of *Salmonella* shedding in horses admitted for routine medical and surgical treatments (Ward et al., 2004).

Transport and/or exercise induce GIT hyperpermeability. Because probiotics have been used to support exercise performance in humans (Pyne et al., 2015), similar interventions have been tested in performance horses. In one study, a probiotic mixture of 5 *Lactobacillus* strains, 2 *Bifidobacterium* strains, and 1 *Streptococcus* strain reduced post-exercise blood lactate concentrations and modified blood and urinary metabolite profiles (Laghi et al., 2018). In another study, a probiotic mixture of 2 *Lactobacillus* strains increased blood oxygen saturation and reduced blood lactic acid concentrations (Zavistanaviciute et al., 2019). Because lactic acid production and accumulation lead to fatigue and reduced performance, probiotics may support athletic performance in horses. Supplementation of an *Aspergillus oryzae* fermentation product prevented leaky gut in horses subjected to transport and exercise (McGilloway et al., 2023b).

## Implications on human health

**Figure 1.**
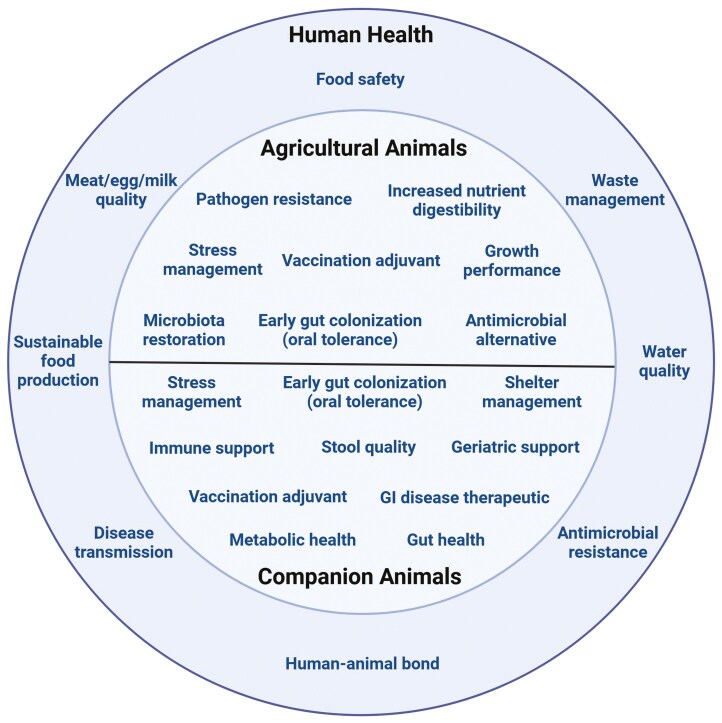
Primary uses of biotics for animal health and production and their impact on human health.

Antimicrobials were used as growth promoters in agricultural animal production for decades, but are now banned by regulatory agencies in many countries due to the increasing global issue of AMR. Moreover, feed antimicrobials used for disease control have been reclassified as Veterinary Feed Directive Drugs by the U.S. Food and Drug Administration (Smith, 2019). From a “One Health” perspective, biotics have been proposed as a valuable tool to improve food safety by reducing the risk of zoonotic bacteria and AMR (Hossain et al., 2017; Joerger and Ganguly, 2017; Monger et al., 2021). Replacing antimicrobials with biotics may reduce colonization or shedding of pathogens (e.g., *Salmonella enterica*) (Barba-Vidal et al., 2017; Kim and Isaacson, 2017; Rodríguez-Sorrento et al., 2020) and reduce antimicrobial-resistant bacterial strains (Stanton and Humphrey, 2011; Achard et al., 2020; Apiwatsiri et al., 2022). While biotics may not completely replace the level of performance produced by antimicrobials, their use leads to a more sustainable and safe food supply. Biotics are known to reduce the presence of antimicrobial residues in food and the environment, improving water quality, waste management, and meat, egg, and milk quality (Fig. 1).

The human-companion animal bond has been researched extensively and shown to improve human health outcomes (e.g., reduction in depression, anxiety, and mortality) (Friedman and Krause-Parello, 2018). Consequently, most of today’s pet dogs and cats are viewed as family members and live inside the home. Unfortunately, this close proximity allows pets to serve as a potential vector for pathogen exposure to humans. Recent studies have shown that co-inhabiting humans and pets share microbiota (Song et al., 2013), that pets may be the source of owner illness (Ngaage et al., 1999; Imanishi et al., 2014), and that AMR in pets may affect human therapeutic options (Ghosh et al., 2011). By reducing pathogen colonization and/or shedding in and around the home and supporting GIT and immune health, reducing the need for antimicrobials, the use of biotics may provide benefits at micro (e.g., individual households, veterinary clinics) and macro (e.g., city wastewater and landfill management) levels in regard to pet ownership.

## Scientific advances and next-generation biotics

Despite the progress that has been made in biotic science over the past few decades, the field has its challenges. Some challenges pertain to product commercialization, quality assurance, and adherence to the variable regulatory standards that exist around the world. Other challenges pertain to the scientific study of biotics and are more relevant in this discussion. A high level of variability is commonly observed regarding biotic responsiveness and efficacy, possibly due to differences among the products tested, the animal populations in question, living conditions, and other factors. The lack of consistency in experimental models and approaches for biotic evaluation is also a likely contributor. Many of these challenges will hopefully be addressed as scientific advancements are made and experimental methodologies are improved.

Advances in microbiome science, the development of more powerful lab assays and equipment, and the adoption of machine learning and artificial intelligence programs that interpret and integrate large, complicated, multi-omic datasets should all improve biotic science. Reduced cost of sequencing and other analytical techniques and development of non-invasive intestinal sampling devices is expected to improve characterization of host GIT microbiota populations, candidate prebiotic substances, and postbiotic preparations, allow for genome mining of candidate probiotics, and provide more accurate assessment of treatment response. Top-down (health-associated microbes, substances, and preparations) and bottom-up (target-based discovery) strategies may be used to identify novel biotics from host organisms, fermented foods, chemically or physically modified substances, or the environment (Cunningham et al., 2021). The continued development and use of in vitro cultivation assays, intestinal organoids, microfluidics, and automated robotics will reduce costs, increase speed and throughput, and improve consistency in the lab, expanding our understanding of treatment responses and pathophysiology of disease (Shah et al., 2016; Chusilp et al., 2020).

**Figure 2.**
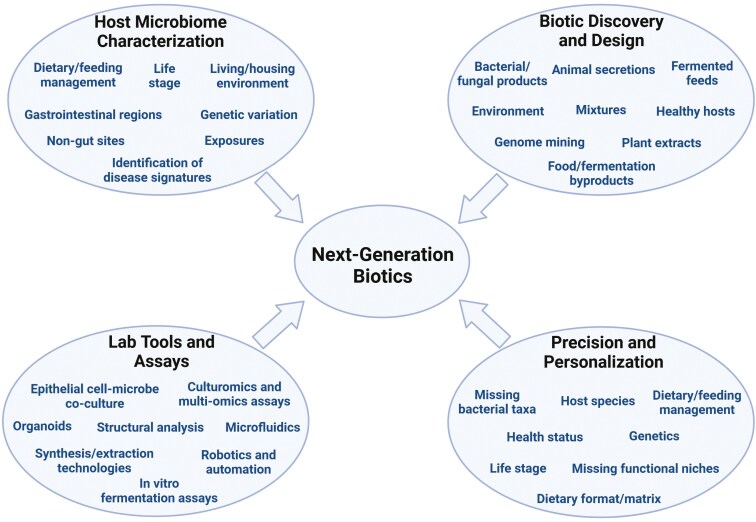
Factors impacting the future of animal biotics.

Scientific advances should lead to more innovative and targeted therapies, including those in the biotic space, resulting in next-generation biotics with greater precision and personalization (Fig. 2). While strategies will certainly be different across agricultural and companion animals, the dietary composition, genetics, health status, commensal microbiome composition and functionality, and other factors within each host species may be used to develop biotics with greater precision. Finally, while biotic strategies will always be largely focused on the GIT, non-GIT targets such as the oral and nasopharyngeal cavities, urogenital tract, skin, and mental wellbeing also will be important targets in the future.

## Conclusions

Biotic substances have been used to promote health of agricultural and companion animals for decades, but responses to treatment have been variable, resulting in a critical need for more robust experimental data to move the field forward. Management of variability, to include use of proper statistical designs and well-powered experiments, choice of appropriate experimental outcomes to be measured, precise definition of the health/production status of the experimental animals being studied, matching the appropriate biotic with desired outcomes, delineation of the host microbiota throughout the experimental period, and improvement in understanding the complicated microbiota-host interactions that are in play will allow for sound interpretation of experimental results, thus allowing for development of more efficacious biotics in the future. Moreover, the use and value of biotics may be viewed beyond the farm, home, or veterinary clinic to the world at large, with implications related to human health and safe food production.

## Abbreviations

AMR: antimicrobial resistance
FMT: fecal microbial transplantation
GIT: gastrointestinal tract
Ig: immunoglobulin
ISAPP: International Scientific Association for Probiotics and Prebiotics
MOS: mannanoligosaccharides
SCFA: short-chain fatty acids
scFOS: short-chain fructooligosaccharides
